# The ototoxic drug cisplatin localises to stress granules altering their dynamics and composition

**DOI:** 10.1242/jcs.260590

**Published:** 2023-07-19

**Authors:** Jack L. Martin, Stephen J. Terry, Jonathan E. Gale, Sally J. Dawson

**Affiliations:** University College London Ear Institute, 332 Gray's Inn Road, London WC1X 8EE, UK

**Keywords:** Cisplatin, Stress granules, Ototoxicity

## Abstract

Cisplatin is an effective platinum-based chemotherapeutic with several side effects, including ototoxicity. Cochlear cells have low rates of proliferation yet are highly susceptible to cisplatin. We hypothesised that cisplatin ototoxicity might be caused by cisplatin–protein interactions rather than cisplatin–DNA interactions. Two known cisplatin-binding proteins are involved in the stress granule (SG) response. SGs are a pro-survival mechanism involving formation of transient ribonucleoprotein complexes during stress. We examined the effects of cisplatin on SG dynamics and composition in cell lines derived from the cochlea and retinal pigment epithelium. Cisplatin-induced SGs are significantly diminished in size and quantity compared to arsenite-induced SGs and are persistent after 24 h recovery. Additionally, cisplatin pre-treated cells were unable to form a typical SG response to subsequent arsenite stress. Cisplatin-induced SGs had significant reductions in the sequestration of eIF4G and the proteins RACK1 and DDX3X. Live-cell imaging of Texas Red-conjugated cisplatin revealed its localisation to SGs and retention for at least 24 h. We show cisplatin-induced SGs have impaired assembly, altered composition and are persistent, providing evidence of an alternate mechanism for cisplatin-induced ototoxicity via an impaired SG response.

## Teaser

**Summary:** Cisplatin causes persistent and aberrant stress granule formation which might contribute to its ototoxicity, a common side effect of chemotherapy treatment for cancer.

## INTRODUCTION

Cisplatin [cis-diammine-dichloroplatinum(II)] is a highly effective, widely used chemotherapeutic for the treatment of solid tumours in a variety of cancers ranging from head and neck to testicular and ovarian cancers. There are, however, several dose-limiting side effects, such as neurotoxicity, nephrotoxicity and ototoxicity. Cisplatin-induced hearing loss is highly dependent on the dose and duration of treatment and occurs in from 20% to 70% of patients, with children showing a greater risk of hearing loss than adults (Li et al., 2004). Hearing loss is initially in the high frequency range but extends to all frequencies and is bilateral and permanent, severely impacting on the quality of life of the patient. The impact is particularly profound for children, potentially affecting speech development and social integration. As cancer survival rates and life expectancy have risen markedly in the past decades, attention is increasingly being paid to the quality of life of patients following treatment (Miller et al., 2016). The need to develop effective therapies to prevent or treat cisplatin-induced ototoxicity is therefore of the utmost importance.

Cisplatin induces DNA damage and cell death in rapidly proliferating tumour cells. It achieves this by binding to DNA via the aquated chloride sites in cisplatin and forming DNA adducts, primarily intra-strand crosslinks. These adducts are recognised by the cell, causing the initiation of several signalling cascades leading to cell cycle arrest and apoptosis (Siddik, 2003). Cochlear cells have low rates of proliferation, with no turnover of the terminally differentiated sensory hair cells, yet the latter are highly susceptible to cisplatin, suggesting that cisplatin ototoxicity might be caused by cisplatin–protein interactions rather than cisplatin–DNA interactions. Although the exact mechanisms behind the ototoxic effects of cisplatin are not fully understood, it is well known that cisplatin-induced ototoxicity is closely related to the accumulation of reactive oxygen species (ROS), endoplasmic reticulum (ER) stress, mitochondrial dysfunction, apoptosis and inflammation-induced damage to the cochlear cells (Kim et al., 2010, 2011). Cisplatin targets at least three major areas in the cochlea; the organ of Corti, spiral ganglion cells and the ion transport epithelium, the stria vascularis (Van Ruijven et al., 2005; Zhang et al., 2020). A characteristic of cisplatin that is highly likely to contribute to its toxicity is its retention within the cochlea, which is in stark contrast to other organs in which cisplatin is eliminated within weeks (Breglio et al., 2017). In 2013, the Steyger laboratory identified several proteins that bind to cisplatin in cochlear cells (Karasawa et al., 2013). Of these proteins, valosin-containing protein (VCP) and heat-shock protein 90 α and β (HSP90α/β) are known to be involved in the stress granule (SG) response (Buchan et al., 2013; Matsumoto et al., 2011).

SGs are membrane-free cytosolic assemblies of messenger ribonucleoproteins (mRNPs) that form in response to the inhibition of translation initiation by environmental stress (e.g. heat, oxidative stress, hypoxia, viral infection and UV). SGs are composed of mRNA, RNA-binding proteins [e.g. cell cycle associated protein 1 (Caprin1), T-cell intracellular antigen 1 (TIA-1), Human antigen R (HuR; also known as ELAVL1) and Ras GTPase-activating protein-binding protein 1 (G3BP1)], 40S ribosomal subunits and mRNA-associated translation initiation complexes (Anderson and Kedersha, 2006). SGs are dynamic structures that quickly form when cells are exposed to stress and normally disperse when the stress is resolved, and normal translation conditions are restored (Kedersha et al., 1999). SG formation is proposed to affect biological reactions in several ways. Firstly, SGs act as an RNA triage centre, sequestering mRNA of housekeeping proteins and prioritising translation of proteins that are involved with the stress response (Anderson and Kedersha, 2008). Secondly, through activation of stress-associated proteins, owing to their concentrating effect. For example, during viral infection, SGs recruit high concentrations of anti-viral proteins, stimulating their activation and enhancing the induction of the innate immune response and restricting virus replication (Rozelle et al., 2014). Finally, through their modulation of signalling pathways by limiting the interactions of sequestered components of signalling pathways, such as receptor for activated C kinase 1 (RACK1), DEAD-Box helicase 3 X-linked (DDX3X), and TNF receptor associated factor 2 (TRAF2) (Arimoto et al., 2008; Samir et al., 2019; Kim et al., 2005).

SG dysfunction has been implicated in the pathogenesis or progression of cancer and neurodegenerative diseases. In neurodegeneration, persistent SGs are thought to act as a focus for pathological protein aggregation (Wolozin and Ivanov, 2019). In cancer, SGs integrate oncogenic signalling and contribute to cancer cell proliferation, invasion, metastasis and drug resistance (Song and Grabocka, 2020). Currently, most SG research has been conducted under acute stress conditions. Although integral to our current understanding of SGs, acute stress bears little resemblance to the chronic nature of diseases in which SGs are known to play a role. Chronic stress can be defined as any stress in excess of 6 h, and initial studies into the dynamics and composition of chronic SGs indicates that with prolonged chronic exposures cells can lose their ‘pro-survival’ phenotype and gain a ‘pro-death’ one in response to chronic stimuli resulting in SG persistence (Reineke and Neilson, 2018).

There is a growing body of work revealing the integral role the SG response plays in the maintenance and protection of hearing. In a previous study, we demonstrated the role SGs play in the response of the cochlea to ototoxic drugs (Towers et al., 2011). Inhibition of SGs with the small-molecule inhibitor ISRIB increased hair cell death in response to aminoglycoside treatment. Conversely, prophylactic induction of SGs with the silvestrol analogue hydroxamate (-)-9 (also known as CR-1-31-B) protected against aminoglycoside-induced hair cell death (Gonçalves et al., 2019). Furthermore, recent work from our laboratory has shown that Caprin1, an RNA-binding protein and key SG component and regulator is necessary for maintenance of auditory function (Nolan et al., 2022).

Considering the above, we hypothesised that the chronic nature of cisplatin treatment and its binding to the SG-associated proteins VCP and HSP90α/β might affect SG dynamics and composition. If so, these changes could explain the mechanism of cisplatin ototoxicity and provide novel targets for the prevention of cisplatin-induced hearing loss. A recent study has shown that U2OS cells form SG-like granules in response to high-dose short-duration cisplatin exposure (Pietras et al., 2022). These granules contained canonical SG components including G3BP1, but lacked mRNA and had reduced eIF4G sequestration. Here, we used clinically relevant concentrations of cisplatin for extended treatment times to explore the effect of cisplatin on SGs in both human retinal pigmented epithelial hTERT RPE-1 cells (RPE), which are similar to the intermediate cells of the stria vascularis, and mouse cochlear-derived UB/OC2 (OC2) cells.

## RESULTS

### Cisplatin induces non-canonical SGs

**Fig. 1.**
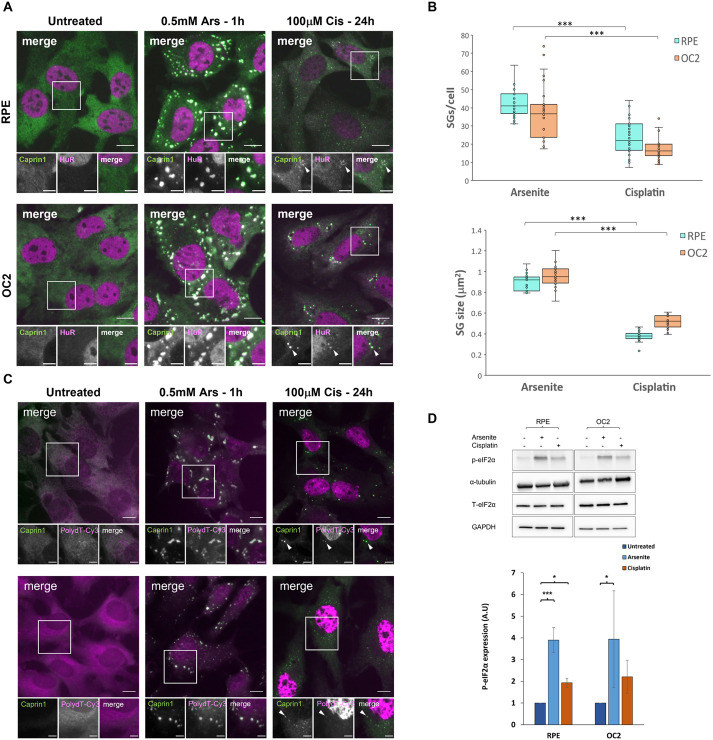
**Cisplatin induces fewer and smaller SGs than sodium arsenite.** (A) RPE and OC2 cells were treated with 0.5 mM sodium arsenite for 1 h or 100 µM cisplatin for 24 h before fixation and immunostaining for the SG markers Caprin1 (green) and HuR (magenta). White arrowheads indicate SGs. Scale bars: 10 µm (main images), 5 µm (smaller images). (B) Quantification of number and size of SGs induced by arsenite and cisplatin treatment. The box represents the 25–75th percentiles, and the median is indicated. The whiskers show the minimum and maximum data values, excluding outliers calculated according to Tukey's method. Result from a minimum of 100 cells (*n*=3, nine coverslips assessed, three from each experiment). ***P<0.001 (unpaired two-tailed Student’s *t*-test). (C) RNA-ImmunoFISH in RPE and OC2 cells with SG marker Caprin1 (green) and polydT-Cy3 (magenta). White arrowheads indicate SGs. Images representative of three experimental repeats. Scale bars: 10 µm (main images), 5 µm (smaller images). (D) Western blot showing the effect of cisplatin on eIF2α phosphorylation and total levels of eIF2α (T-eIF2α), and densitometry of western blot bands for RPE and OC2 cells. Error bars represent s.d. (*n*=3). *P<0.05, ***P<0.001 (unpaired two-tailed Student’s *t*-test). A.U., arbitrary units.

To investigate the effect of cisplatin on SG dynamics and the role of the SG response in the development of cisplatin ototoxicity, we first sought to establish a protocol for use with the mouse cochlear-derived OC2 cell line and the hTERT RPE cell line that would replicate as closely as possible the clinical concentrations and chronic nature of cisplatin treatment in chemotherapy. We treated cells with 20, 50 or 100 µM cisplatin for 16 or 24 h. The SG markers Caprin1 and HuR were used to assess SG formation and determine that the minimum concentration for induction of SGs in both cell lines was 100 µM (Fig. S1). Given that there was a more robust response at 24 h than 16 h, 100 µM cisplatin for 24 h was used to induce SGs in subsequent experiments. When cisplatin-induced SGs were compared to SGs produced upon sodium arsenite treatment, a well-established inducer of oxidative stress, cisplatin induced far fewer and significantly smaller (both *P*<0.001) SGs in both RPE and OC2 cells (Fig. 1A; quantification in Fig. 1B). SGs are typically defined by the presence of SG markers like Caprin1 and/or G3BP1 but also mRNA. To assess whether cisplatin-induced granules contain poly(A) mRNA RNA-ImmunoFISH was performed on arsenite- and cisplatin-treated cells. As can be seen from Fig. 1C, cisplatin-induced granules do contain poly(A) mRNA (white arrowheads) and can therefore be defined as SGs. The phosphorylation status of eIF2α, a key event in the initiation of SG formation, was determined by western blotting and shows that cisplatin causes a significant (*P*<0.05) 2-fold increase in the levels of phosphorylated (p)-eIF2α in RPE cells and a non-significant 2-fold increase in OC2 cells compared to that seen on untreated cells (Fig. 1D), whereas total levels of eIF2α remined the same. However, this level of eIF2α phosphorylation was lower than the 4-fold increase produced by sodium arsenite in both cell lines (Fig. 1D).

### Cisplatin-induced SGs are persistent

**Fig. 2.**
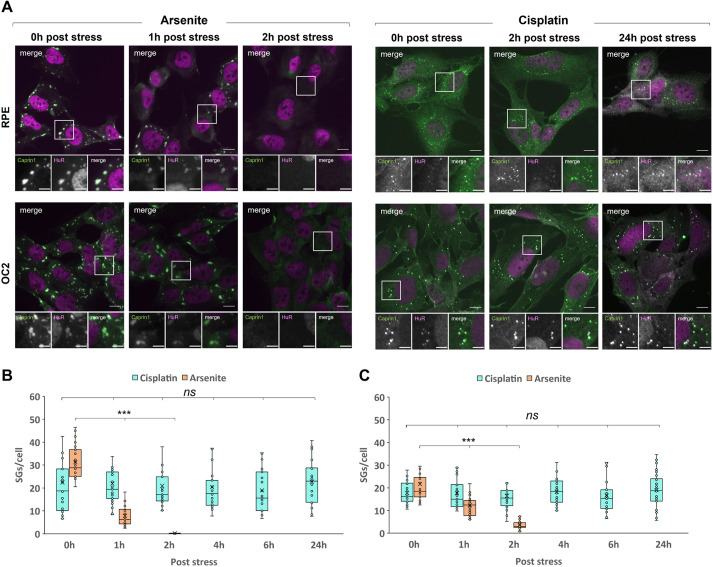
**Cisplatin-induced SGs are persistent.** (A) RPE and OC2 cells were treated with 0.5 mM sodium arsenite for 1 h or 100 µM cisplatin for 24 h. Medium was removed, and cells washed before fresh medium was added and cells were allowed to recover. Cells were fixed after the indicated recovery times and stained for the SG markers Caprin1 (green) and HuR (magenta). Scale bars: 10 µm (main images), 5 µm (smaller images). (B,C) Graphs show quantification of SGs per cell for RPE (B) and OC2 (C) cells immediately after stress and up to 24 h recovery. The box represents the 25–75th percentiles, and the median is indicated. The whiskers show the minimum and maximum data values, excluding outliers calculated according to Tukey's method. *n*=3. ***P<0.001; ns, not significant (one-way ANOVA with Tukey’s multiple comparisons).

Canonical SGs are transient aggregates that disassemble and clear once the stress has been removed or resolved. Persistent SGs that do not clear, however, have been implicated in multiple neurodegenerative diseases and have been referred to as pathological SGs (Wolozin and Ivanov, 2019). Given the assembly differences between arsenite-induced SGs (Ars-SGs) and cisplatin-induced SGs (Cis-SGs), we next investigated the clearance of Cis-SGs. As expected, Ars-SG clearance was immediate and rapid; at 1 h after removal of arsenite the number of SGs had decreased by 95% and 82% for RPE (Fig. 2B) and OC2 (Fig. 2C) cells, respectively. In stark contrast to the rapid disassembly of Ars-SGs, there was no significant change in the number of Cis-SGs per cell at all time points measured post stress. Remarkably, even at 24 h post-cisplatin treatment, SGs still remained (Fig. 2A). The effects of cisplatin on cell toxicity were also examined; as shown in Fig. S6, this reveals that cisplatin treatment results in fewer cells after 24 h compared to untreated cells. Whether the reduced cell count is due to a reduction in cell division or cell death remains to be determined.

### Cisplatin-treated cells have an impaired SG response to arsenite

**Fig. 3.**
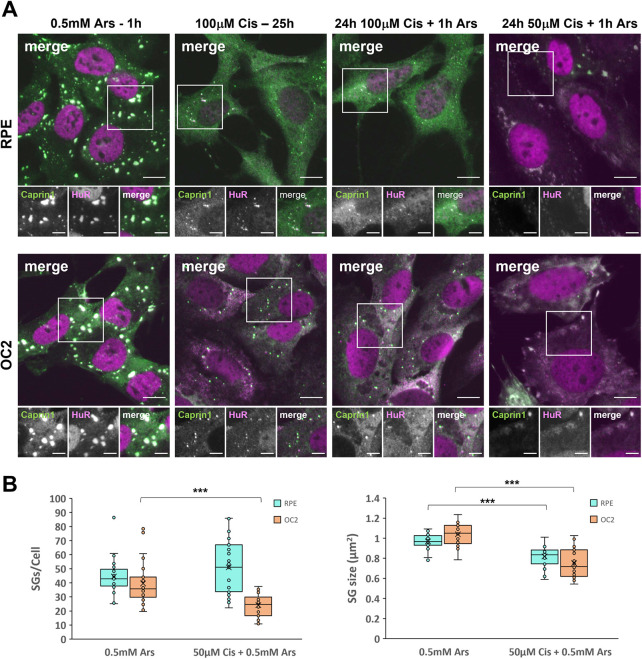
**Cisplatin-treated cells exposed to additional arsenite stress have an impaired SG response.** (A) RPE and OC2 cells were treated with either sodium arsenite for 1 h, 100 µM cisplatin for 25 h and either 100 µM or 50 µM cisplatin for 24 h before the co-application of 0.5 mM sodium arsenite for the last hour. Scale bars: 10 µm (main images), 5 µm (smaller images). (B) Quantification of SGs per cell and SG size for cells treated with arsenite and 50 µM cisplatin prior to arsenite. The box represents the 25–75th percentiles, and the median is indicated. The whiskers show the minimum and maximum data values, excluding outliers calculated according to Tukey's method. *n*=3. ***P<0.001 (unpaired two-tailed Student’s *t*-test).

The ototoxicity associated with cisplatin can develop several months after treatment (Kolinsky et al., 2010) as cisplatin is retained much longer in the cochlea than other tissues (Breglio et al., 2017). Given the persistence of cisplatin-induced SGs, we questioned how cisplatin-treated cells would respond to a subsequent stress. To examine this, cells were treated with either 50 µM or 100 µM cisplatin for 24 h prior to the addition of 0.5 mM arsenite for 1 h. Cells were fixed and immuno-stained for Caprin1 and HuR. Interestingly, cells previously treated with 100 µM cisplatin, which produce fewer and smaller SGs (see Fig. 1), were unable to generate typical Ars-SGs in response to subsequent arsenite stress. As can be seen in Fig. 3A, comparing 100 µM cisplatin treatment with and without subsequent arsenite treatment, there was no change in the size or quantity of the SGs, nor any additional SG formation in either cell line in response to this subsequent stress. Furthermore, in cells treated with 50 µM cisplatin, a concentration insufficient to induce SGs alone (Fig. S1), and subsequently challenged with arsenite there are clear differences between the SGs formed compared to canonical arsenite-induced SGs (Fig. 3A,B) suggesting this dose of cisplatin still influences SG assembly. OC2 cells exposed to cisplatin for 24 h formed far fewer SGs in response to arsenite than ‘naïve’ cells treated with arsenite only. There was a significant 59% reduction (*P*<0.001) in the number of SGs per cell and a 28% reduction in the average size of those SGs compared to arsenite-only SGs (Fig. 3B). In cisplatin pre-treated RPE cells, there was no significant difference in the numbers of SGs per cell compared to that seen with arsenite treatment alone. However, a frequency histogram reveals that the two populations of cells responded to the additional arsenite in different ways (Fig. S2); 55% of cisplatin pre-treated RPE cells form more than 50 SGs per cell compared to 27% for arsenite alone. The analysis for the size of these SGs, showed a significant 16% reduction (*P*<0.001) in the average size of these SGs (Fig. 3B).

### Cisplatin-induced SGs contain eIF3η but lack eIF4G

**Fig. 4.**
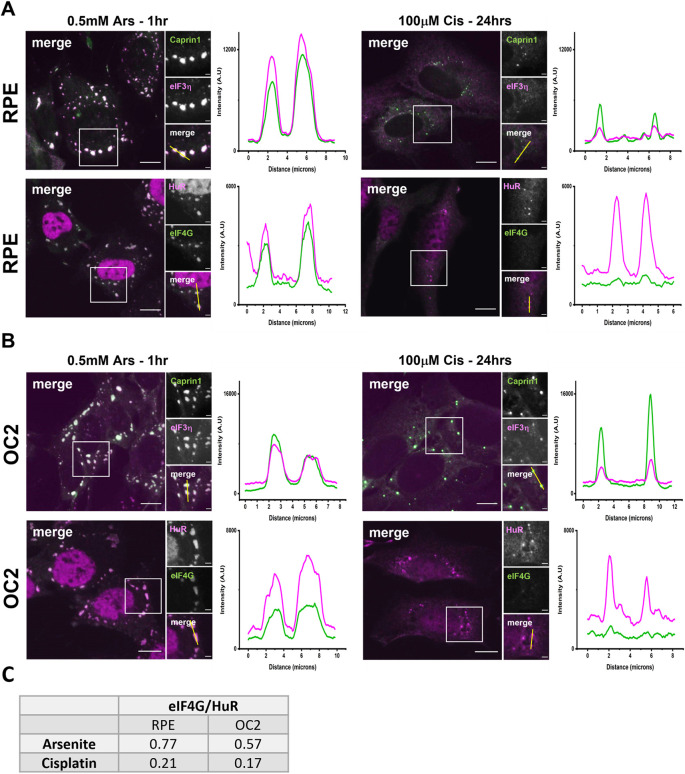
**Cisplatin-induced SGs contain eIF3η but have a marked reduction in sequestration of eIF4G.** (A) RPE and (B) OC2 cells were treated with 0.5 mM sodium arsenite for 1 h or 100 µM cisplatin for 24 h before fixation. They were then immunostained for Caprin1 (green) and eIF3η (magenta), or HuR (magenta) and eIF4G (green). Fluorescence intensity line plots showing the signal intensity along the corresponding yellow line (in small merge panel) indicate the changes in relative sequestration of canonical SG proteins. Scale bars: 10 µm (main images), 5 µm (smaller images). (C) Quantification of eIF4G sequestration to RPE and OC2 cells in response to arsenite and cisplatin. A line was drawn through five SGs per image and the fluorescence intensity along the line was measured. Using Origin™ 2021 software, the area under the peaks was measured, giving the ratio of eIF4G to HuR. SGs quantified=135, *n*=3 experiments.

Canonical SGs contain components of the eukaryotic translation initiation complex (Kedersha et al., 2005), including eIF3η and eIF4G. We examined the sequestration of these components in Cis-SGs by immuno-staining arsenite- and cisplatin-treated cells with antibodies for eIF3η, eIF4G, Caprin1 and HuR. In both cell types eIF3η was sequestered to Cis-SGs, but to a lesser degree than for Ars-SGs (Fig. 4A,B). However, there was a marked decrease in the sequestration of eIF4G to Cis-SGs. The presence of eIF4G was barely detectable in the immunofluorescence images for both RPE cells (Fig. 4A) and OC2 cells (Fig. 4B). The fluorescence intensity plots, however, show small peaks in the intensity of eIF4G at the same points as the HuR peaks, indicating that there is sequestration of eIF4G, but that it is severely reduced. Quantification of eIF4G (Fig. 4C) shows a reduction in eIF4G sequestration in both cell lines in response to cisplatin.

### Cisplatin-induced SGs have reduced sequestration of DDX3X

**Fig. 5.**
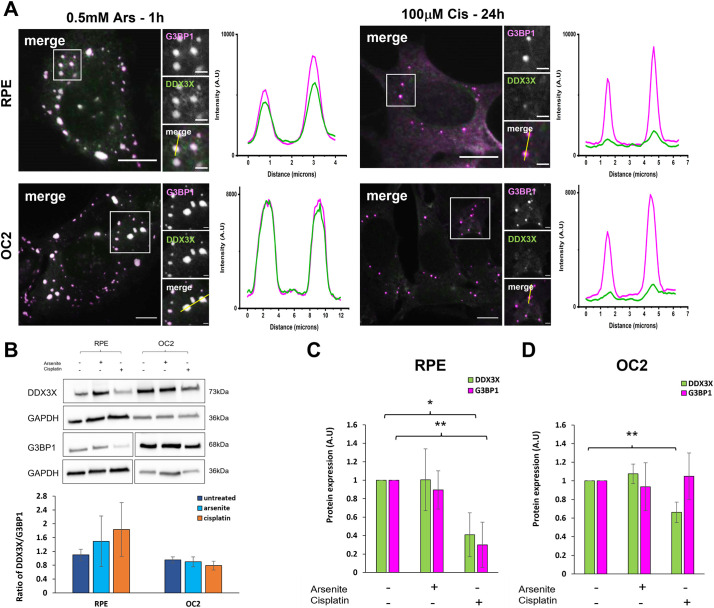
**Cisplatin-induced SGs fail to sequester DDX3X effectively.** (A) Representative widefield immunofluorescent images of RPE and OC2 cells treated with either 0.5 mM sodium arsenite for 1 h or 100 µM cisplatin for 24 h before fixation. Cells were immunostained for G3BP1 (magenta) and DDX3X (green). Fluorescence intensity line plots for G3BP1 and DDX3X were measured from the corresponding yellow line (in small merge panel). Scale bars: 10 µm (main images), 2 µm (smaller images). (B) Western blot of DDX3X and G3BP1 in untreated, arsenite and cisplatin treated cells and ratio of DDX3X to G3BP1 levels from densitometry. GAPDH was used as loading control for densitometry analysis of RPE (C) and OC2 (D) cells. Error bars represent s.d., *n*=4 experiments. *P<0.05, **P<0.01 (unpaired two-tailed Student’s *t*-test). A.U., arbitrary units.

SGs can act as signalling hubs, sequestering proteins involved in signalling pathways and thereby influencing cell fate. One recently identified signalling protein, namely DDX3X, acts as a cellular life or death decision point by regulating the formation and activation of the NLRP3 inflammasome, an activity which is inhibited by its sequestration to SGs (Samir et al., 2019). To investigate the capacity of Cis-SGs to sequester DDX3X, arsenite- and cisplatin-treated cells were immunostained with antibodies for DDX3X and the SG marker G3BP1 (Fig. 5A). In agreement with other studies, DDX3X was sequestered to Ars-SGs in both cell lines, as can be seen from the strong colocalisation in the images and corresponding fluorescence intensity line plots. In stark contrast, Cis-SGs had substantially reduced sequestration of DDX3X. To examine the possibility that the reduced sequestration of DDX3X to Cis-SGs is a consequence of a reduction in the levels of DDX3X, we performed western blots on untreated, arsenite- and cisplatin-treated cells (Fig. 5B). In RPE cells, there was a significant 70% and 82% reduction in the levels of DDX3X and G3BP1, respectively, compared to that seen in untreated cells (*P*<0.05 and *P*<0.001, respectively). In OC2 cells, there was no significant change in the level of G3BP1, whereas there was a 33% reduction in the level of DDX3X (*P*<0.05, Fig. 5D). The levels of G3BP2 were also examined in response to cisplatin and were found to be reduced in OC-2 cells in response to cisplatin but were unaffected in RPE cells (Fig. S7).

### Cisplatin-induced SGs have reduced RACK1 sequestration and RACK1 localises to P bodies in OC2 cells

**Fig. 6.**
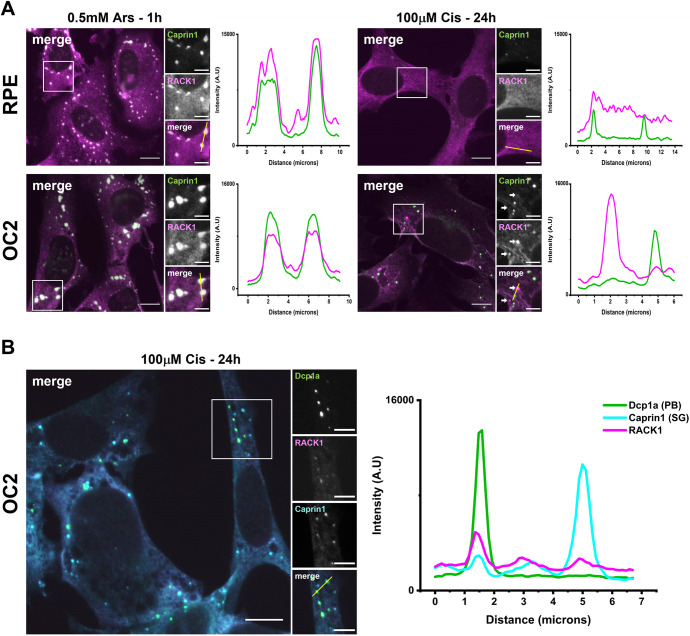
**Cisplatin-induced SGs have reduced RACK1 sequestration and in OC2 cells RACK1 is localised to PBs.** (A) RPE and OC2 cells were treated with either 0.5 mM sodium arsenite for 1 h or 100 µM cisplatin for 24 h before fixation. Cells were then stained for Caprin1 (green) and RACK1 (magenta). (B) Cisplatin treated OC2 cells were then stained for the PB marker Dcp1a (green), RACK1 (magenta) and Caprin1 (cyan). Fluorescence intensity line plots show the signal intensity along the corresponding yellow line (in small merge panel). Images representative of *n*=3. Scale bars: 10 µm (main images), 5 µm (smaller images). A.U., arbitrary units.

Given the reduced sequestration of DDX3X to Cis-SGs, we next examined the sequestration of another key signalling protein, RACK1. SG formation represses pro-apoptotic MAPK signalling through recruitment of RACK1 to SGs (Arimoto et al., 2008). To investigate the capacity of Cis-SGs to sequester RACK1, both arsenite- and cisplatin-treated cells were immunostained with antibodies for RACK1 and Caprin1 (Fig. 6A). Consistent with previous work (Arimoto et al., 2008), RACK1 was found to be sequestered to Ars-SGs in both cell lines. However, in cisplatin-treated cells there was a reduction in the sequestration of RACK1 to SGs in both cell lines. Unexpectedly, in OC2 cells there were RACK1 puncta that did not show colocalization with Caprin1 puncta (Fig. 6A, white arrows). The RACK1 peak in the fluorescence intensity plot did not overlap with the peak in the Caprin1 puncta. The size, quantity and location of RACK1 puncta in relation to the Caprin1 puncta were reminiscent of the P bodies (PBs) observed in cisplatin-treated cells. Immunostaining cisplatin-treated cells for the PB marker Dcp1a in addition to Caprin1 and RACK1 confirmed that RACK1 localised to PBs in cisplatin-treated OC2 cells (Fig. 6B).

### Live-cell imaging reveals that cisplatin localises to SGs

**Fig. 7.**
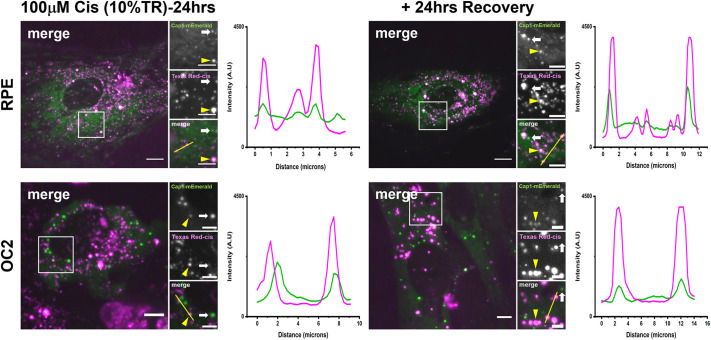
**Live-cell imaging reveals cisplatin–TR is localised to SG and is retained 24 h post stress.** RPE and OC2 cells stably expressing Caprin1–mEmerald were treated with 100 µM cisplatin, 10% of which was cisplatin–TR for 24 h. After 24 h, the medium was changed, and cells were imaged on a spinning disc confocal. Cells were then allowed to recover for 24 h before being imaged again. Yellow arrowheads in all images indicate colocalization of Caprin1 and cisplatin–TR. White arrows indicate Caprin1 puncta that are not localised with cisplatin–TR. Fluorescence intensity line plots show the signal intensity along the corresponding yellow line (in small merge panel). Images representative of *n*=3. Scale bars: 10 µm (main images), 5 µm (smaller images). A.U., arbitrary units.

We next used Texas Red-conjugated cisplatin (cisplatin–TR) to investigate the localisation of fluorescently labelled cisplatin in these cell lines. Ascertaining the distribution of cisplatin within the cell would help in determining the action site of cisplatin (i.e. whether it is acting by directly interfering with the assembly of these SGs, or indirectly by compromising the activity of one of the previously identified cisplatin-binding proteins). To examine the localisation of cisplatin, we generated two new cell lines, RPE and OC2 cells stably expressing Caprin1–mEmerald (Figs S3, S4) and treated these cells with 100 µM cisplatin, 10% of which was cisplatin–TR. After 24 h of cisplatin exposure, both cell lines formed SGs, as can be seen by the Caprin1–mEmerald puncta (Fig. 7). In both cell lines there was strong colocalisation of cisplatin–TR with Caprin1–mEmerald. However, there are also a number of Texas Red puncta that are distinct from Caprin1 puncta, and Caprin1 puncta that are not localised with Texas Red. For both cell lines, there was no change in the localisation of cisplatin–TR with SGs at 24 h after recovery.

## DISCUSSION

Recent data has established that SGs play an important role in auditory protection including from ototoxic aminoglycoside antibiotics (Nolan et al., 2022; Gonçalves et al., 2019). Cisplatin is another common ototoxic agent and there is evidence that it might interact directly with SG proteins (Karasawa et al., 2013). Here, we investigated the effects of cisplatin on the SG response and potential implications for ototoxicity. Firstly, we found that 24 h of 100 µM cisplatin was sufficient to induce a SG response, in both RPE and OC2 cells (Fig. 1A; Fig. S1). However, SGs induced by cisplatin were significantly smaller and fewer than those induced by arsenite (Fig. 1B). Cisplatin-associated stress (ROS production, ER stress etc.) induces SG formation. The reduced size and quantity of these non-canonical Cis-SGs could be a result of cisplatin binding to nascent SGs and affecting their correct assembly. SGs contain mRNA, as well as canonical SG markers like Caprin1 and G3BP1. Here, we used RNA-ImmunoFISH (Fig. 1C) to determine that cisplatin-induced granules do contain poly(A) mRNA and that these granules are indeed SGs. Previous work has shown that 250 µM cisplatin treatment for 4 h resulted in granules that lacked mRNA (Pietras et al., 2022). One potential explanation for this difference is the different treatment regimes used. Here, we used a lower, more clinically relevant concentration over a longer time to induce SGs. To ascertain whether these smaller Cis-SGs have different properties to typical SGs, we examined the composition of Cis-SGs and found that, although they contain eIF3η, they have severely reduced levels of eIF4G, almost below the level of detection for both cell lines (Fig. 4). A key step in typical SG assembly involves G3BP1 binding to the translation initiation factor eIF4G (Yang et al., 2019); therefore, granules lacking eIF4G might be expected to have impaired or altered function. In agreement with this hypothesis, we found that the sequestration of two key signalling proteins DDX3X and RACK1 was significantly reduced in both cell lines (Figs 5A and 6A). One of the ways that SGs effect protection is by sequestering certain signalling proteins. Sequestration of DDX3X to SGs prevents the formation and activation of the NLRP3 inflammasome and the subsequent release of pro-inflammatory cytokines IL-1β and IL-18, thereby preventing pyroptosis (Samir et al., 2019). We have shown that Cis-SGs have reduced sequestration of DDX3X, and consequently unsequestered DDX3X will be available to facilitate the assembly and activation of the NLRP3 inflammasome, which requires further investigation. Indeed, previous work has documented that cisplatin has effects on inflammation in both the kidney and cochlea. In renal tubular epithelia, cisplatin causes an increase in NLRP3, ASC (also known as PYCARD), caspase-1 and IL-1β expression (Li et al., 2019). Cisplatin has also been shown to induce activation of perivascular resident macrophage-like melanocytes in the stria vascularis as well as an increase in the levels of IL-1β and caspase-1 in the cochlea (Kim et al., 2015; Zhang et al., 2020). The reduction in DDX3X sequestration by the aberrant/non-canonical Cis-SGs in the kidney and the cochlea could explain the increase in inflammasome components and inflammatory cytokines. Anti-inflammatory drugs like dexamethasone have been shown to offer protection against cisplatin ototoxicity to varying degrees, although like other prospective treatments, it has only been shown to confer partial protection in clinical trials (Marshak et al., 2014). Our findings identify a potential novel target for pharmaceuticals. To our knowledge, inhibition of the NLRP3 inflammasome has not yet been explored as a treatment against cisplatin ototoxicity. It has, however, been shown to attenuate cisplatin-induced renal fibrosis (Li et al., 2019). To determine whether the reduced sequestration of DDX3X was due to a cisplatin induced reduction in its levels, western blots were performed. As can be seen from Fig. 5B–D, in RPE cells there is a reduction in both DDX3X and G3BP1. Importantly, the level of DDX3X is greater than G3BP1, which indicates that the reduced DDX3X sequestration to SGs is not simply a result of a reduction in DDX3X. Furthermore, in OC2 cells there is only a small reduction in DDX3X and no reduction in G3BP1, which supports the conclusion that the reduced sequestration of DDX3X is not due to reduction in its expression. Additionally, the lack of change in G3BP1 levels in OC2 cells indicates the reduced size and quantity of Cis-SGs is not due to a reduced pool of G3BP1, further supporting the conclusion that Cis-SGs have impaired assembly. Levels of the closely related G3BP2 (Matsuki et al., 2013) are unaffected in RPE cells, suggesting that this is not a general translational effect (Fig. S7).

Autophagy has been shown to be partly responsible for the clearance of SGs (Buchan et al., 2013) and multiple studies have demonstrated that enhancing autophagy attenuates cisplatin-induced ototoxicity (Liu et al., 2019; Pang et al., 2019; Yang et al., 2018). It is reasonable, in the light of the data presented herein, to assume that part of the protective effect of autophagy activators results from the clearance of these persistent SG–cisplatin aggregates. A possible approach to attenuate cisplatin ototoxicity would be to use NLRP3 inhibitors to reduce the damage caused by inflammation in conjunction with autophagy activators to clear the persistent SGs. However, other studies have shown that excessive autophagy accelerates cisplatin-induced cochlear cell death (Yin et al., 2018). The current balance of evidence is in favour of inducing autophagy to attenuate cisplatin ototoxicity, but this has not been established. Additionally, it is essential that any treatment that prevents cisplatin ototoxicity should not interfere with its tumour-killing activity. For ototoxicity, this might be avoided by local intratympanic application of the inhibitor either into the middle ear or directly on to the round window rather than by systemic application.

Having observed the effect of cisplatin on SG assembly and persistence together with data from Karasawa et al. (2013) suggesting that cisplatin binds SG proteins, we investigated whether cisplatin itself could interact with SGs. By using cisplatin–TR, we discovered that cisplatin does localise to SGs in live cells. Previous work has shown that cisplatin can concentrate in MED1 nuclear condensates, contributing to cisplatin pharmacodynamics (Klein et al., 2020), but to our knowledge this is the first time that the localisation of cisplatin with SGs has been observed. The localisation of cisplatin–TR at SGs raises the possibility that the reduced size and quantity of these SGs, as well as the reduced sequestration of DDX3X and RACK1 might be a consequence of direct or local interference by cisplatin with the nascent assembling SG. This does not exclude the possibility that cisplatin is also affecting the activity of one of the previously identified cisplatin-binding proteins that are involved in SG assembly and clearance (Karasawa et al., 2013). It is possible that the reason cisplatin is localised to SGs is because of its binding with VCP and/or HSP90α/β; any effect of cisplatin on their activity remains to be determined. The persistence of Cis-SGs and the localisation of cisplatin–TR to these SGs, even 24 h after recovery, is a possible explanation for the retention of cisplatin in the cochlea. The localisation of cisplatin–TR to SGs also supports the hypothesis that lower concentrations of cisplatin affect the fusion of Ars-SGs. In both cell lines, we observed SGs that lack cisplatin–TR and cisplatin–TR puncta that were not localised with SGs. A simple explanation for finding SGs that do not contain cisplatin–TR is that only 10% of the cisplatin was cisplatin–TR. Therefore, it is possible that these SGs contain untagged cisplatin. The cisplatin–TR puncta that are distinct from SGs, could be either PBs or possibly lysosomes.

SGs are transient condensates that disassemble and clear once the stress has been resolved or removed (Kedersha et al., 2005). Persistent SGs have been linked to multiple neurodegenerative diseases and are thought to act as a focus for the aggregation of disease-related proteins (Wolozin and Ivanov, 2019). When we examined the clearance of Cis-SGs, we found that they were extremely stable, persisting for at least 24 h post stress (Fig. 2). The persistence of these Cis-SGs indicates they might be irreversible, permanently stalled at an early point in SG assembly. Recent studies have shown that the composition of persistent SGs differs from that of transient SGs. Persistent SGs induced by chronic nutrient starvation lack 18S rRNA, RACK1 and RPS6 (Reineke et al., 2018). The composition of SGs under chronic stress conditions is an emerging area of SG research, and more work is needed to understand the differences between various chronic stresses. This present work adds to a growing body of work on the dynamics and composition of SGs formed in response to chronic stress. In addition to the impaired SG response to cisplatin and the change in SG composition, we have shown that cisplatin-treated cells are unable to mount a SG response to further insults (Fig. 3A). This inability to mount a further SG response to arsenite could sensitise cochlear and other cells to future or further insults by compromising the protective function of SGs. This could be a key underlying cause of the ototoxicity and could also provide an explanation for the delayed onset of cisplatin-induced hearing loss in some patients (Kolinsky et al., 2010). It can take several months for hearing loss to develop, in which time the patient would likely experience multiple insults above the SG-inducing threshold. The reduced size and increased quantity of Ars-SGs in RPE cells previously exposed to 50 µM cisplatin (Fig. 3B) suggests concentrations of cisplatin that are insufficient to induce SGs alone can have an impact on the fusion of smaller SGs into larger mature SGs induced by a further stimulus. An unexpected but interesting result from our work was the localisation of RACK1 to PBs in cisplatin-treated OC2 cells (Fig. 6B). RACK1 is thought to be exclusive to SGs, and to our knowledge, this is the first time that RACK1 has been shown to localise to PBs. PBs are thought to act as storage sites for translationally repressed mRNAs and inactive mRNA decay enzymes, although their exact role remains controversial. This discovery raises the possibility of a novel function of PBs, where in situations that compromise the signalling activity of SGs, PBs might be able to compensate by sequestering signalling proteins themselves. Further work is required to determine whether RACK1 sequestration by PBs silences it, reducing downstream signalling and apoptosis.

Recently, Pietras and colleagues have shown that a 4 h treatment of 250 µM cisplatin is sufficient to induce SG-like granule formation in cancer-derived cell lines (Pietras et al., 2022). In our non-cancerous cell lines, one of which is cochlear derived, 250 µM cisplatin for 4 h was insufficient to induce SG formation (Fig. S5). Chronic stress can be defined as a stress in excess of 6 h. In the cochlea, cisplatin is known to be retained indefinitely (Breglio et al., 2017) and can therefore be considered a chronic stress. Additionally, concentrations of cisplatin higher than 100 µM have been shown to be less ototoxic (Ding et al., 2011). Crucially, we have examined the SG response to extended cisplatin exposure at low concentration, in order to reproduce the chronic nature of chemotherapy treatment at clinically relevant doses. An important finding in our study is that Cis-SGs contain poly(A) mRNA and can be classified as SGs, albeit ones that have impaired assembly, increased persistence and altered composition.

**Fig. 8.**
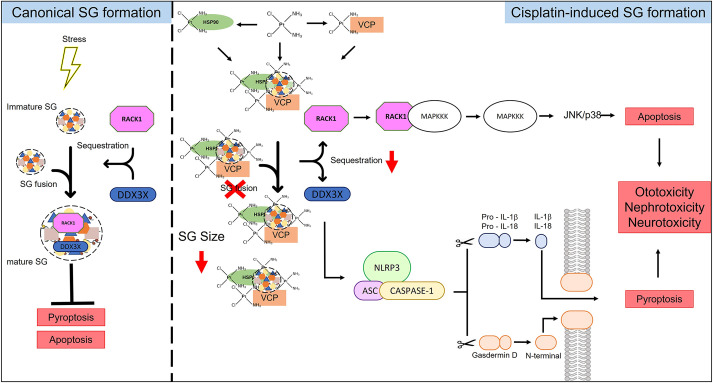
**Proposed model of cisplatin toxicity in non-tumour cells.** In canonical SG formation, stress causes the inhibition of translation initiation, polysome disassembly and SG formation. SGs sequester RACK1 and DDX3X, preventing their participation in their respective signalling pathways, promoting cell survival. In cisplatin-induced SG formation, cisplatin localises to SGs, either by directly binding to SGs or by binding with VCP and HSP90. This accumulation of cisplatin at SGs impairs their assembly, alters their composition and affects their persistence. These Cis-SGs have greatly reduced sequestration of signalling proteins RACK1 and DDX3X. These signalling molecules are therefore available to participate in their respective signalling pathways, resulting in the induction of pyroptosis and/or apoptosis.

Taken together, our study has revealed that cisplatin localises to SGs, and causes their impaired assembly, increased persistence and altered composition, leading to the formation of what could be termed non-canonical SGs. Furthermore, cells that have been exposed to cisplatin are unable to form SGs in response to additional stress. If this scenario were replicated in the cochlea, it could sensitise cochlear cells to further insults by compromising their protective function. The aberrant/non-canonical SGs induced by cisplatin could underlie the susceptibility of cochlear cells to cisplatin, but this requires further investigation *in vivo*. They might also provide an explanation for the retention of cisplatin in the cochlea and represent several novel targets for the prevention of cisplatin ototoxicity. These findings might also have wider implications for cisplatin-resistant tumours by increasing our understanding the non-DNA effects of cisplatin treatment. We propose a model of cisplatin toxicity based on these findings (Fig. 8) in which aberrant SGs and their altered composition play a role in the development of cisplatin-induced hearing loss.

## MATERIALS AND METHODS

### Cell lines

UB/OC-2 (OC2) cells, derived from the Immortomouse™ (Rivolta et al., 1998) were cultured at 33°C under 5% CO_2_ atmosphere in Dulbecco's modified Eagle's medium (DMEM) supplemented with 10% fetal bovine serum (FBS) and 50 units/ml γIFN, as described previously (Towers et al., 2011). hTERT RPE-1 (RPE; ATCC, CRL-4000) cells were cultured at 37°C under 5% CO_2_ in DMEM-F12 (Gibco) supplemented with 10% FBS, 1% penicillin/streptomycin, 1% GlutaMAX (Gibco) at 37°C and 5% CO_2_.

### DNA constructs

We used a pLenti-EFS-mEmerald-MCS-IRES-G418, 3rd generation lentivirus vector allowing the co-expression of a green fluorescent protein (mEmerald) and antibiotic selection marker conferring resistance to Neomycin (G418) from a single EFS promoter (Human eukaryotic translation elongation factor 1 α1 short form) made by VectorBuilder. The pLenti-EFS-mEmerald-Caprin1-IRES-G418 (containing Caprin1 with a mEmerald N-terminal tag), was made by using the NEB HiFi assembly method following the manufacturer's protocol and online primer design tool (https://nebuilder.neb.com/), with modifications outlined as follows: pLenti-EFS-Emerald-MCS-IRES-G418 was digested with restriction enzymes (Xho1 and Nde1) (NEB) and gel purified. PCR of Caprin1 was performed using a plasmid template (Caprin1 plasmid provided by John W. Schrader, University of British Columbia, Vancouver, Canada; Solomon et al., 2007) and primers containing 25 bp of homology to pLenti-EFS-mEmerald-MCS-IRES-G418 forward (5′-CTGTACAAGGGTGGCGGAGGCTCTCTCGAGGGATCCATGCCCTCGGCC-3′) and reverse (5′-CCGGGTCTAGAGTCGACCTGCAGCATATGGGATCCTTAATTCACTTGCTGAGTGTTC-3′). Vectors were transformed in NEB stable *Escherichia coil* (NEB) and grown at 30°C to minimise recombination events. Vectors were DNA sequenced via sanger sequencing method (Eurofins-MWG), prior to viral transduction.

### Production of stable cell lines

Cell lines were made by viral transduction using lentiviruses. For viral transduction, lentiviral packing vector pLenti-EFS-mEmerald-Caprin1-IRES-G418 was co-transfected with packaging system pMD2.G containing VSVg and psPax2 (Addgene plasmids #12259 and #12260, deposited by Didier Trono) into HEK 293T cells (a gift from the laboratory of Ulrike Eggert, Randall Centre for Cell & Molecular Biophysics, King's College London, UK) using Linear 25,000 MW Polyethleimine (PEI) (Polysciences) at ratio of 6:1 PEI:DNA. 48 h after transfection supernatants were collected, clarified by centrifugation (200 ***g*** for 5 min), filtered (0.45 μm), and used to infect RPE and OC2 cells at multiplicity of infection (MOI) <1, cell lines were selected after a further 48 h post infection with G418 (Life Technologies) at final concentrations 800 μg/ml, respectively. Cell lines were grown on maintenance concentration of or 400 μg/ml G418.

### Antibodies

Primary antibodies used were against: Caprin1 (Proteintech 15112-1-AP, 1:500), HuR (Santa Cruz Biotechnology SC-5261, 1:500), G3BP1 (Santa Cruz Biotechnology SC-365338, 1:500), G3BP1 (Abcam ab556574, 1:500), Dcp1a (Abcam ab57654, 1:500), eIF3η (Santa Cruz Biotechnology SC-137214, 1:100), eIF4G (Cell Signaling Technology 2498S, 1:100), DDX3X (Proteintech 11115-1-AP, 1:100), RACK1 (BD Transduction Laboratories 610177, 1:100), GAPDH (Proteintech 60004-1-Ig, 1:50,000) T-eIF2α (Cell Signaling Technology 9722S, 1:1000) and eIF2α [pSer52] (Enzo BML-SA405-0100, 1:1000).

Second antibodies used were: goat-anti-rabbit IgG conjugated to Alexa Fluor 633 (Invitrogen A21070, 1:500), goat-anti-mouse IgG conjugated to Atto-550 (Sigma, 1:1000), goat-anti-mouse IgG2a conjugated to Alexa Fluor 488 (Invitrogen A21131, 1:1000), goat-anti-mouse IgM conjugated to Alexa Fluor 633 (Invitrogen A21046, 1:500), goat-anti-rabbit IgG conjugated to Alexa Fluor 546 (Invitrogen A11035, 1:1000), DAPI (chromatin labelling, 1 mM 1:1000), donkey-anti-mouse-HRP (Jackson ImmunoResearch 715-035-150, 1:10,000), donkey-anti-rabbit-HRP (Jackson ImmunoResearch 711-035-152, 1:10,000).

### Drug treatments

Cisplatin concentrations were based on a recent study in mice, where the concentrations of cisplatin were chosen to mimic clinical doses. In that study 3 mg/kg body weight of cisplatin was administered in a cyclic drug administration protocol (Gersten et al., 2020), giving a peak blood concentration 171 µM (assuming mouse blood volume of 1.46 ml, average weight 25 g, and formula weight of cisplatin 301.1 g/mol).

Cisplatin (Sigma) was reconstituted in saline at a concentration of 3 mM prior to each experiment and added to cells to reach the desired final concentration. When recovery periods were applied, the medium containing cisplatin was removed, and cells were rinsed and incubated with new cisplatin-free medium for the specified period before fixation. For live imaging of fluorescently tagged cisplatin, cells stably expressing Caprin1–mEmerald were plated on µ-slide angiogenesis slides (Ibidi) and incubated for a minimum of 16 h prior to addition of cisplatin. Texas Red-conjugated cisplatin (Ursa Biosciences) was reconstituted in saline and added to non-tagged cisplatin to reach 10% of the total cisplatin. After addition of total cisplatin, cells were incubated for 24 h, the medium was removed, cells were washed and cisplatin-free medium was added prior to imaging on a Zeiss Axiovert 200 microscope. After imaging, cells were allowed to recover for 24 h before re-imaging. For arsenite-induced stress, cells were incubated for 1 h with 0.5 mM sodium arsenite (Sigma) before fixation. When recovery periods were applied, the medium containing sodium arsenite was removed, and cells were rinsed and incubated with new arsenite-free medium for the specified period before fixation.

### Immunofluorescence and RNA-immunoFISH

Cells were fixed in 4% paraformaldehyde for 15 min and then blocked/permeabilised for 30 min in blocking buffer containing 0.3% Triton-X (Sigma) and 10% goat serum (Gibco), before incubation with primary antibodies (in blocking buffer) for 1 h at room temperature. After washing with PBS, cells were incubated with the secondary antibodies and DAPI (in blocking buffer) for 1 h at room temperature. Coverslips were then washed and mounted on slides using Fluoromount-G (SouthernBiotech). Images were acquired on the Zeiss Axioimager M2 microscope.

For RNA-immunoFISH, cells were permeabilised with −20°C methanol for 10 min and rinsed twice with 2xSSC at 25°C. Hybridisation was performed at 43°C for 14 h in the dark in RNA hybridisation mixture containing 25% (v/v) formamide, 200 ng/μl salmon sperm DNA, 5× Denhardt's solution, 50 mM sodium phosphate pH 7, 1 mM EDTA, 2× SSC and 200 ng/μl of polydT-5′ probe (Eurofins). Primary antibody detection was performed following the above immunofluorescence protocol.

To exclude crosstalk between channels, samples were imaged on a Zeiss 880 confocal microscope with emission filtering that excluded crosstalk between Cy3 [excitation (Ex) 561 nm, emission (Em) 566–608 nm] and Alexa Fluor 633 (Ex 633 nm, Em 641–723 nm), see Fig. S8A. Additionally, RNA-ImmunoFISH was performed in the absence of the anti-Caprin1 antibody and punctate cytoplasmic staining of poly(A) mRNA was observed (Fig. S8B).

### Immunofluorescence quantification

Using ImageJ, a line was drawn through five SGs per image and the fluorescence intensity along the line was measured. The intensity values were exported to Origin 2021 software and the area of the peaks measured, giving the ratio of eIF4G to HuR.

### SG quantification

The ‘SG counter’ Fiji plugin (Ann Sablina, Lomonosov Moscow State University, Russia; https://imagej.nih.gov/ij/plugins/stress_granule_counter/SG_counter.class) was used to determine the number and size of SGs. For all SG quantifications, the Caprin1 signal was used.

### Western blotting

Protein levels for DDX3X, G3BP1 and p-eIF2a were assessed by western blotting. After incubation, cells were lysed in 1.5× Laemmli buffer containing protease inhibitors (Roche). Protein concentrations were determined with a DC protein assay kit (Bio-Rad). Proteins were separated with Bio-Rad mini protean TGX 8–16% gels and afterwards transferred onto nitrocellulose membranes. Membranes were blocked in 5% milk powder in TBS with 0.1% Tween 20 (TBST) for 1 h and incubated with primary antibodies overnight at 4°C. Membranes were washed with TBST before incubation with secondary antibodies for 1 h at room temperature. Band intensity was detected with ECL prime (Cytiva) using a Syngene G:box and quantified with ImageJ. GAPDH was used as a loading control. Raw western blot data is provided in Fig. S9.

### Statistics

Statistical analyses were performed using an unpaired two-tailed Student's *t*-test or one-way ANOVA with Tukey's multiple comparisons correction (in SPSS software). Significance is denoted as *P<0.05, **P<0.01 and ***P<0.001.

## Supplementary Material

10.1242/joces.260590_sup1 Supplementary information
