# Supplementary material for: The ototoxic drug cisplatin localises to stress granules altering their dynamics and composition
Source: J Cell Sci. 2023 Jul 19;136(14):jcs260590. doi: 10.1242/jcs.260590 (PMC10399988; doi:10.1242/jcs.260590)
Supplement: Supplementary information [file joces-136-260590-s1.pdf]

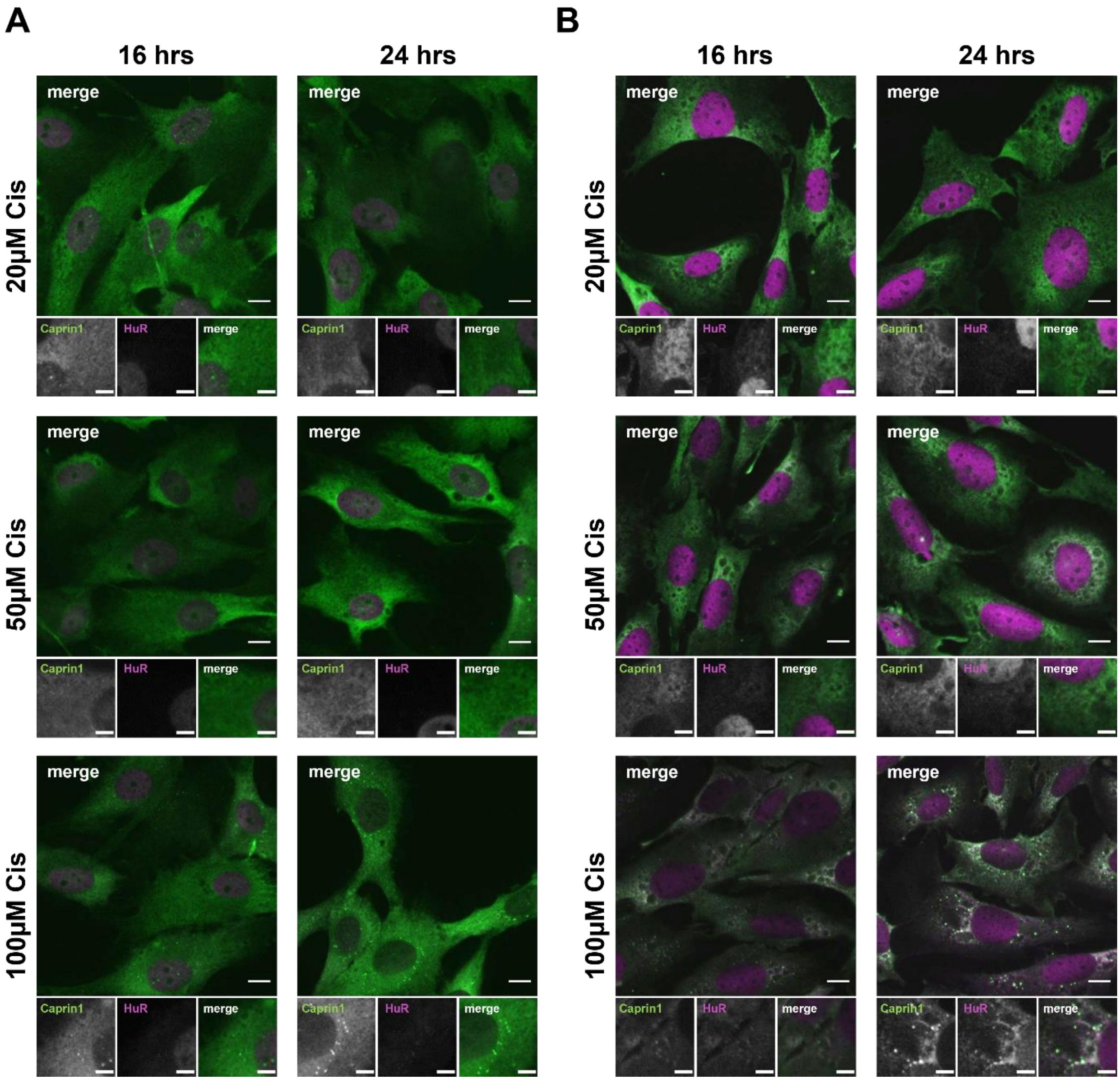

Fig. S1. Treatment of cell lines over time with varying concentrations of cisplatin. (A) OC2 and (B) RPE cells were treated for 16 or 24h with either 20, 50 or 100µM cisplatin before fixation and immuno-staining with Caprin1 and HuR.

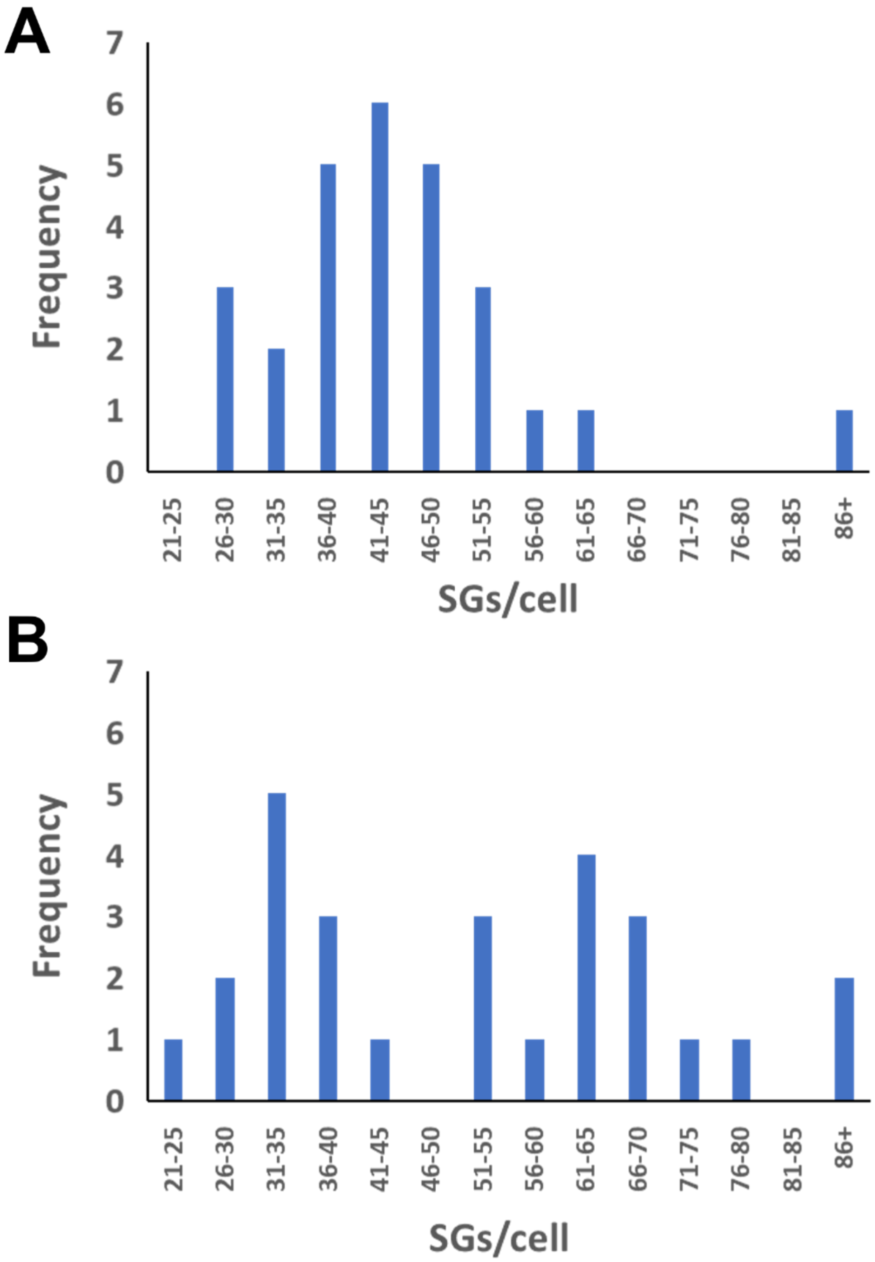

Fig. S2. Frequency distribution for SG/cell in RPE cells. Frequency distribution for SGs/cell in RPE cells treated with either arsenite (A) for 1h or pre-treated with 50μM cisplatin for 24h before arsenite (B).

Journal of Cell Science • Supplementary information

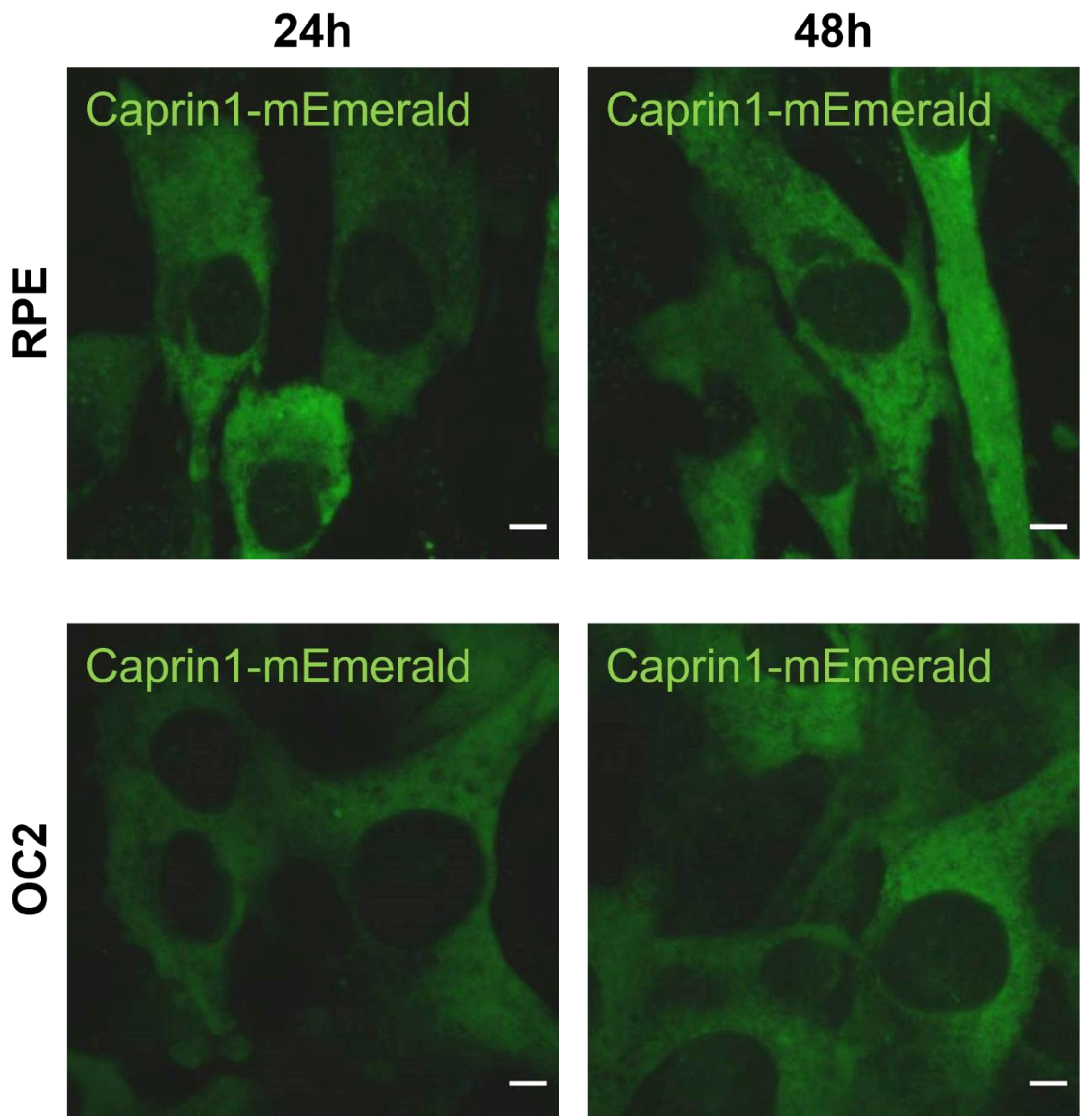

Fig. S3. Caprin1mEmerald cell lines untreated at 24h and 48h. RPE and OC2 cells stably expressing Caprin1-mEmerald at 24h and 48h.

Journal of Cell Science • Supplementary information

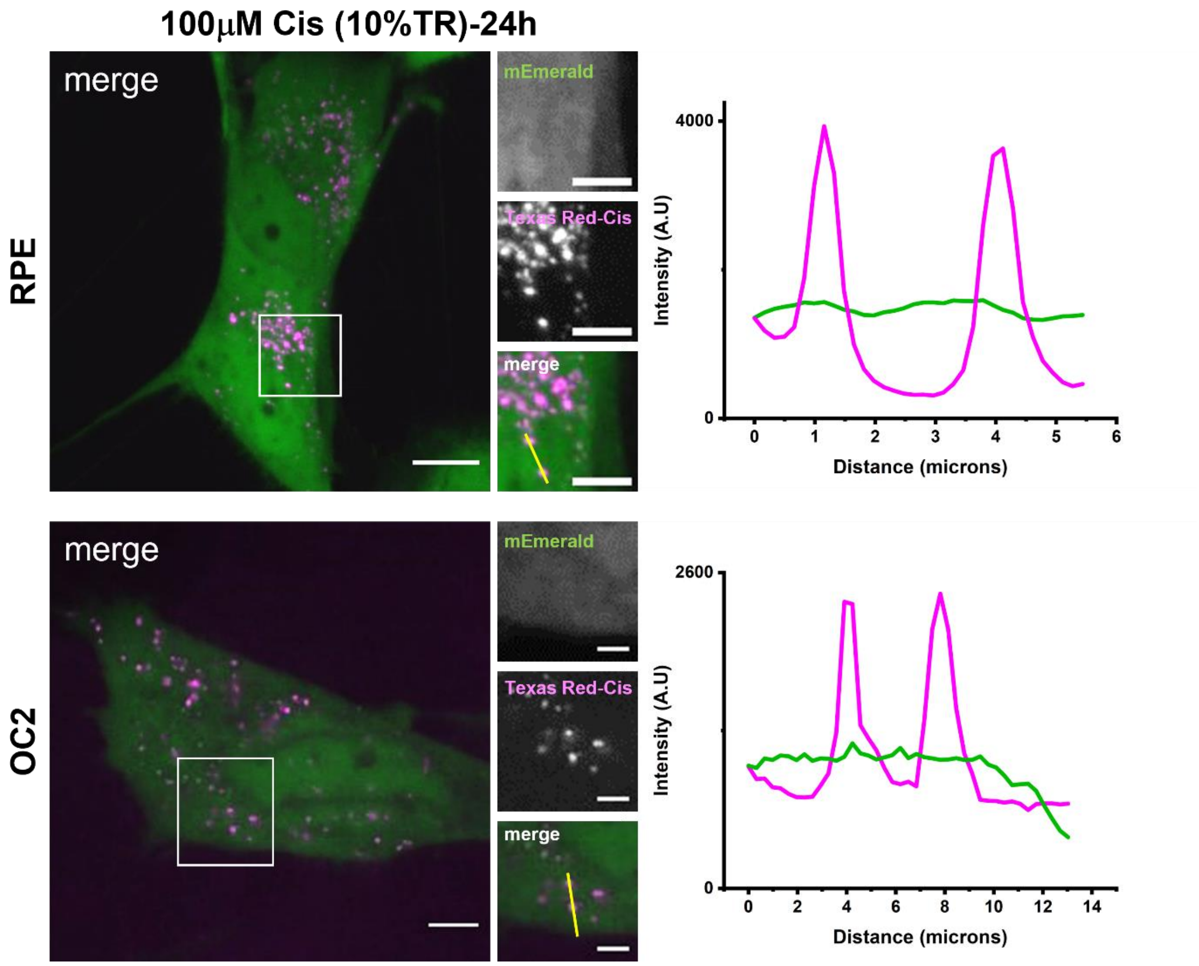

Fig. S4. mEmerald cell lines treated with Texas Red cisplatin. RPE and OC2 cells stably expressing mEmerald treated with 100µM cisplatin, 10% of which was Texas-red cisplatin for 24h. Profile plots show the signal intensity along the corresponding yellow line. Scale bars =10µm in large image and 5µm in zoomed.

Journal of Cell Science • Supplementary information

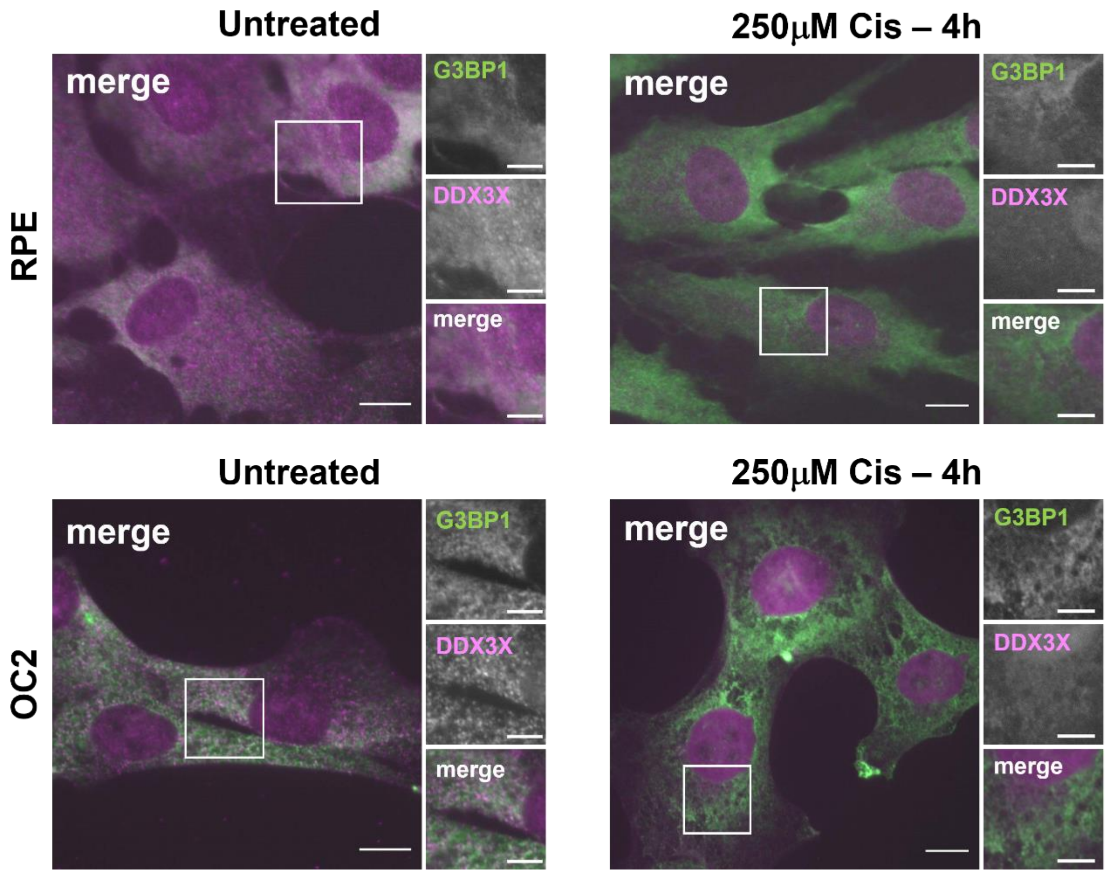

Fig. S5. RPE and OC2 cells treated with 250μM cisplatin for 4h. RPE and OC2 cells were treated with 250μM cisplatin for 4h before fixation and immuno-staining with G3BP1 and DDX3X.

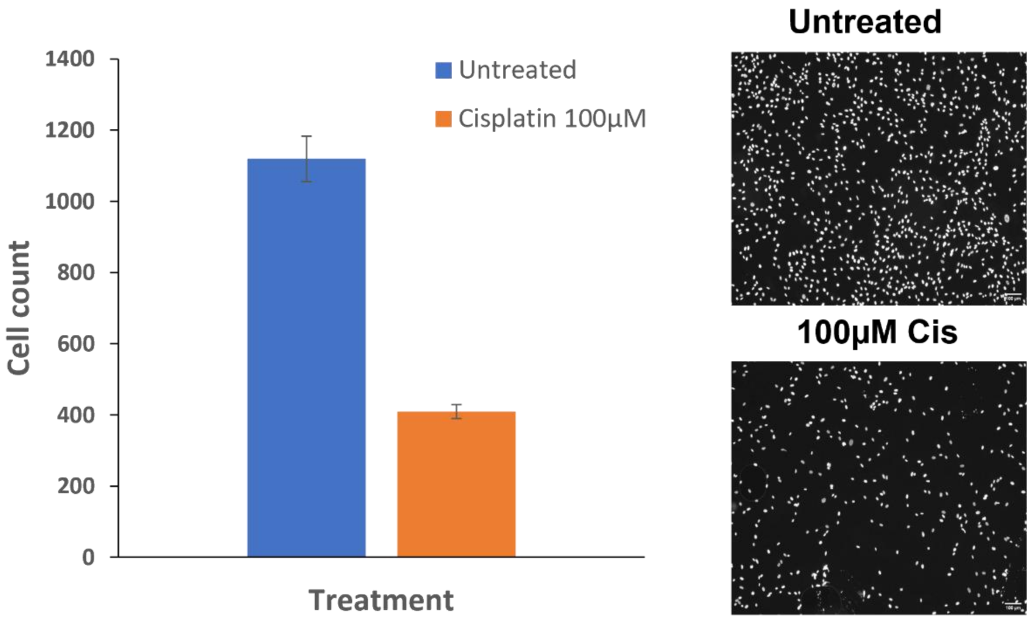

Fig. S6. Cell count of cisplatin treated RPE cells. RPE cells were treated with 100mM cisplatin for 24h before fixation. Cells were stained with DAPI and then 10x images per cover slip were collected and compared to untreated cells. N = 3.

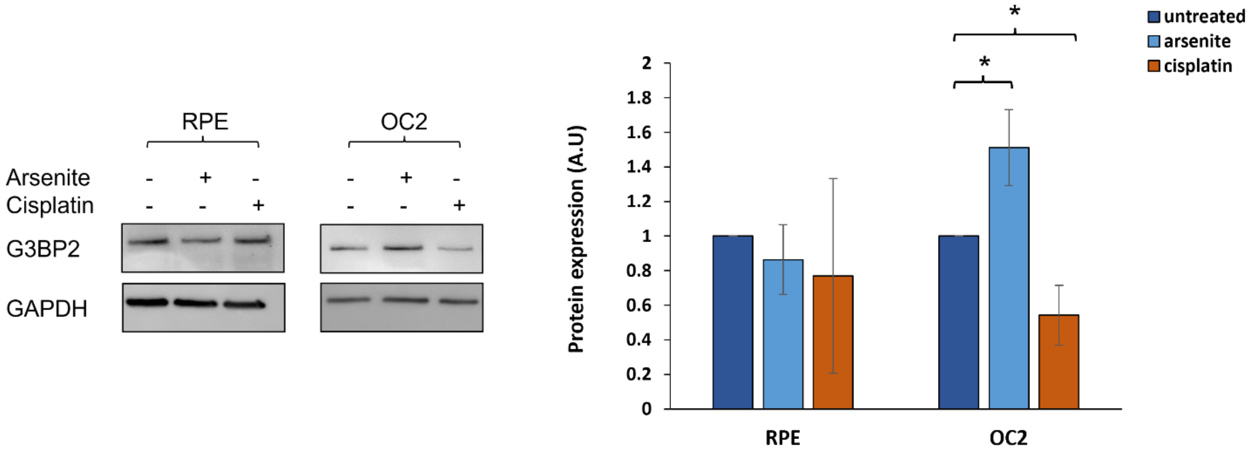

Fig. S7. Levels of G3BP2 in RPE and OC2 cells. Western blot of G3BP2 in untreated, arsenite and cisplatin treated cells. GAPDH used as loading control for densitometry analysis. Error bars represent SD. *= $p<0.05$. N=3.

Journal of Cell Science • Supplementary information

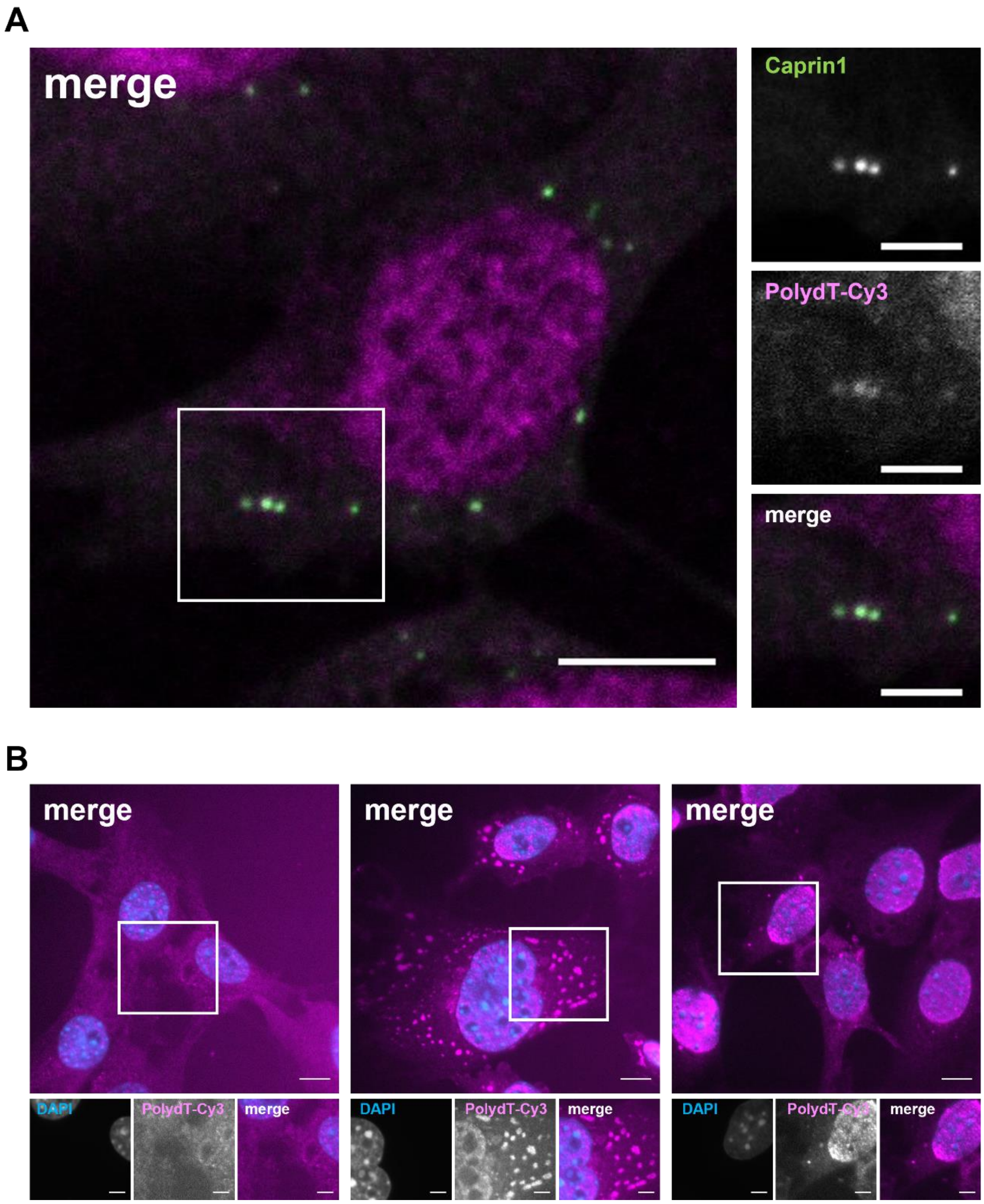

Fig. S8. RNA-ImmunoFISH images of cisplatin treated OC2 cells. (A) Cisplatin treated cells were imaged using a Zeiss 880 confocal microscope with Ex/Em filters that excluded crosstalk between AlexaFluor633 (641-723) and Cy3 (566-608). Scale bar in main image = 10μm, in small images = 5μm. (B) Cells were either left untreated or treated with 0.5mM sodium arsenite for 1h or 100μM cisplatin for 24h before RNA-ImmunoFISH. Immuno was performed without Caprin1 or GaR-633. Scale bar in main image = 10μm, in small images = 5μm.

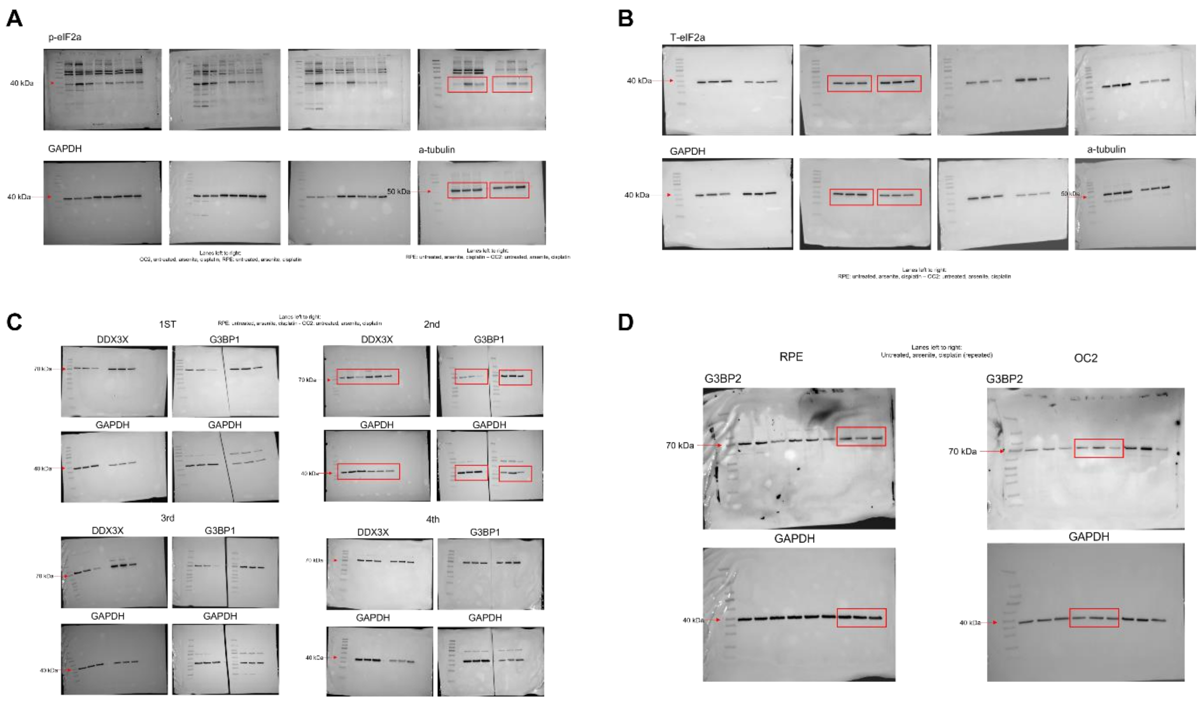

Fig. S9. Blot transparency. Raw western blot data is provided for each corresponding figure. (A) Raw blots for p-eIF2α with loading control GAPDH and α-tubulin. (B) Raw blots for total eIF2α with loading controls GAPDH and α-tubulin. (C) Raw blots for DDX3X and G3BP1 with loading control GAPDH. (D) Raw blots for G3BP2 and loading control GAPDH. Red boxes indicate data used in figures.

Journal of Cell Science • Supplementary information
